# Vehicle Localization Using Doppler Shift and Time of Arrival Measurements in a Tunnel Environment

**DOI:** 10.3390/s22030847

**Published:** 2022-01-22

**Authors:** Rreze Halili, Noori BniLam, Marwan Yusuf, Emmeric Tanghe, Wout Joseph, Maarten Weyn, Rafael Berkvens

**Affiliations:** 1 IMEC-IDLab, University of Antwerp, Sint-Pietersvliet 7, 2000 Antwerp, Belgium; rreze.halili@uantwerpen.be (R.H.); noori.bnilam@uantwerpen.be (N.B.); maarten.weyn@uantwerpen.be (M.W.); 2 IMEC-WAVES, Ghent University, Technologiepark-Zwijnaarde 126, 9052 Gent, Belgium; marwan.yusuf@ugent.be (M.Y.); emmeric.tanghe@ugent.be (E.T.); wout.joseph@ugent.be (W.J.)

**Keywords:** RF, localization, GNSS, tunnel, C-ITS

## Abstract

Most applications and services of Cooperative Intelligent Transport Systems (C-ITS) rely on accurate and continuous vehicle location information. The traditional localization method based on the Global Navigation Satellite System (GNSS) is the most commonly used. However, it does not provide reliable, continuous, and accurate positioning in all scenarios, such as tunnels. Therefore, in this work, we present an algorithm that exploits the existing Vehicle-to-Infrastructure (V2I) communication channel that operates within the LTE-V frequency band to acquire in-tunnel vehicle location information. We propose a novel solution for vehicle localization based on Doppler shift and Time of Arrival measurements. Measurements performed in the Beveren tunnel in Antwerp, Belgium, are used to obtain results. A comparison between estimated positions using Extended Kalman Filter (EKF) on Doppler shift measurements and individual Kalman Filter (KF) on Doppler shift and Time of Arrival measurements is carried out to analyze the filtering methods performance. Findings show that the EKF performs better than KF, reducing the average estimation error by 10 m, while the algorithm accuracy depends on the relevant RF channel propagation conditions and other in-tunnel-related environment knowledge included in the estimation. The proposed solution can be used for monitoring the position and speed of vehicles driving in tunnel environments.

## 1. Introduction

The Global Navigation Satellite System (GNSS) is the most widely used technology for vehicle positioning or localization. However, GNSS requires an unobstructed line of sight to satellites, which makes it impossible to use in obstructed areas, such as tunnels, urban canyons, and other indoor environments [1]. To overcome these obstructions, a number of studies have considered the use of communication technologies opportunity signals as an alternative or a complement to GNSS regarding localization.

As concluded in our previous work [2], the localization accuracy obtained when using vehicular communication technologies depends on the bandwidth, received signal-to-noise ratio (SNR), frequency, maximum transmitted power, geometric position of ground stations, etc. This can be a challenging situation since these technologies are designed to provide quality communication links and not accurate localization. In addition, when the environment is indoors, such as a tunnel, this becomes even more complex since the signal propagation analysis used for communication in a tunnel are not straightforward.

### 1.1. Propagation in Tunnel Environments

Radio wave propagation in a tunnel strongly depends on the frequency [3]. From one point of view, above a certain frequency, a tunnel-like environment can behave as a waveguide, guiding the radio signal with the tunnel-defined structure and extending the communication range since the attenuation is much lower compared to free space, outdoor, or other indoor environments [1]. On the other hand, the signal might suffer from strong periodic spatial fading phenomena and have multipath propagation when it is obstructed and blocked from the line-of-sight (LOS) transmission. In this situation, the transmission distance is reduced.

There has been a huge effort in terms of accurate mathematical modeling and measuring campaigns to predict radio wave propagation in tunnels to ensure the good quality of communication links and increase the channel capacity. These are useful for vehicular networks, train applications, and even service and surveillance missions in both military and civilian contexts [4,5,6,7,8]. Deterministic channel models for tunnels include waveguide models, ray tracing models, and numerical methods for solving Maxwell’s equations in tunnel environments [9]. These methods suffer from large computational complexity and incomplete description of the propagation environment (scatterers, mobility, traffic, etc.). On the other hand, analytical stochastic models that are obtained from measurements in real traffic conditions describe the specific environment with less computational cost [9]. As the propagation is influenced by many factors (e.g., tunnel geometry, obstacles, nodes setup, traffic), measurements in practical scenarios are required to characterize and model the propagation in tunnels [10].

### 1.2. Localization In-Tunnel Environments

When it comes to the localization topic, propagation path losses and fadings, specifically generation and detection of the known-geometry fading structures are useful for elaboration of coverage maps, localization, and navigation inside tunnels [11,12,13,14]. An appropriate choice of the antenna positions and periodic patterns, regarding fading over distance, can enable one-dimensional localization by detecting the changes in the slope of the received signal fadings, precisely by having the number of maxima and minima encountered on the received signal wavelength [10,11,12,14]. However, the traffic condition disturbs the propagating modes, making the pseudo-periods less clear than in empty tunnels [10], and this causes localization errors. The presence of traffic, the number and size of vehicles increase the signal attenuation and fluctuations too, especially close to vehicles [15]. According to [16], in a typical situation when the distance is greater than 100 m, the localization accuracy will be degraded.

In addition, different localization algorithms and technologies are considered. Laser, simultaneous localization, and mapping (SLAM) and Global Positioning System (GPS) are commonly adopted for the localization of vehicles, and when GPS is not available in tunnels, studies [17,18,19] make use of an environment static map to improve the SLAM matching confidence. Inertial navigation systems (INS) are self-contained dead reckoning, which use accelerometers and different sensors to provide dynamic information through measurements from inertial measurement units (IMU). However, errors, scale factors, and nonlinearity in sensor readings cause errors accumulating with time [20,21]. To bound the accumulation of errors, INS is coupled with GNSS signals [20], yet in GNSS-denied environments, accuracy improvement is possible for very short GNSS signal outages. According to [22], when traveling at 100 km/h, the dead-reckoning system can estimate the vehicle’s location for less than 30 s with an error bound of 10 m. Radar technology, camera systems, laser scanners, and odometer sensors are also mentioned in different studies [23]. However, because of noise and errors in sensors data, limited performance in environments with low visibility, high cost, line of sight requirements between anchors and agents, environmental pollution with radar signal, cumulative errors, etc., the accuracy of the estimated position degrades the longer the estimation runs [4,21,24].

Therefore, Vehicular-to-Everything (V2X) communication technology is also exploited to mitigate the positioning error when the GNSS signals are missing. Cellular network technologies (UMTS, LTE, 5G) and wireless local network technologies (WiFi/WLAN, Bluetooth, Ultra Wide Band (UWB), Zigbee, RFID, Dedicated Short Range Communication (DSRC)) are considered. The provided accuracy depends on the number of base stations and their positions, the bandwidth dedicated for localization, propagation conditions, etc. While coverage, deployment cost, and latency are other impact factors when choosing between these cellular and wireless networks [25]. In addition, advanced positioning techniques, such as proximity, triangulation of the received signals, and fingerprinting, together with different filtering techniques, are used to improve in-tunnel localization accuracy.

The performance limits of localization in outdoor urban and highway with LTE networks are simulated in [26], using different bandwidth ranges from 20 to 100 MHz. The study indicates that the bandwidth significantly enhances the positioning performance of each network deployment. Similarly, the hybridization of GNSS and simulated LTE signals to analyze localization performance in an urban scenario is performed in [27]. Different studies require the synchronization and coverage of a number of LOS transmitter nodes and consider the difference between Time of Arrival (ToA) measurements to perform the receiver node position estimation using multilateration or trilateration [28,29,30].

Opportunistic use of vehicular ad-hoc networks (VANETs) has been proposed by many studies as the smart solution to realize connections between vehicles and road infrastructure components required for many services, including accurate positioning. Kalman filter (KF) and Extended Kalman filter (EKF) fed by measured Received Signal Strength (RSS), ToA, Time Difference of Arrival (TDoA) [22,31], and Angle of Arrival (AoA) [32], have been investigated to improve positioning performance. A combination between vehicles Vehicle-to-Vehicle (V2V) and Vehicle-to-Infrastructure (V2I) communication, sensors, and map information with the use of Unscented Kalman filter (UKF) for heading estimation and a particle filter fed by V2V signal strength measurements is investigated in [33] to improve localization performance. Particularly, ref. [34] presents a driverless experiment and addresses the use of IEEE 802.11p V2V communication to provide inter-vehicle cooperation in order to ensure a safe distance between vehicles.

RSS, ToA, TDoA, AoA, and their combination are considered in different studies [35,36,37,38]. According to [35], the combination of TDoA and AoA provides higher positioning accuracy than RSS, but the cost is very high; thus, RSS is seen as a good candidate to meet the requirements of precision considering the hardware cost. In [39], it has been noticed that accuracy can be improved when modeling the localization uncertainty propagation, and considering this result in motion planning can increase reliability.

### 1.3. Contribution

Adding more components to a tunnel for the purpose of vehicle localization, using different technologies from the current solutions used for communication, and adding more processing units to vehicles, causes more expenses and increases the system’s complexity. Moreover, this approach adds the need for compatibility between different standards and technologies and causes different delays, dependencies, problems, and uncertainties [40,41]. Thus, in this work, we use our previous studies and analysis on the non-stationary V2I channel in the frequency band of LTE-V [10] to address vehicle localization in tunnels and to show preliminary results on this matter.

The provided solution uses existing V2I communication architecture, so it uses one single transmitter. First, we give attention to our result of the measurement campaign in the Bervend tunnel in Belgium. Then, we focus on the use of the developed algorithm that combines the GNSS initial position with the Doppler shift and ToA measurements to have accurate vehicle location estimation and speed. To the best of the authors’ knowledge, most of the consulted studies and most of the localization solutions are based on simulations. In addition to this, most of the solutions need a dense network topology, rely on both V2I and V2V communication, and sometimes require dedicated positioning protocols or positioning reference signals [16,22,27,31,32]. Therefore, to have vehicle’s location and speed information in a tunnel environment, we propose, I. a novel solution algorithm that makes use of Doppler shift measurements obtained in the LTE-V frequency band from one single transmitter; II. the proposed solution algorithm can also combine Doppler shift and ToA measurements received from the same transmitter; III. the proposed solution algorithm performs physical layer signal processing of the existing C-ITS communication signals to monitor the vehicle’s position and speed. To further improve the algorithm performance, we use EKF and/or KF to mitigate the channel propagation effects. The results indicate that the proposed algorithm accuracy depends on the channel propagation knowledge, considered parameters, and motion model. The proposed solution can reduce the C-ITS infrastructure cost and communication, as it can have an impact on the safety, reliability, and efficiency of C-ITS applications and services.

The rest of the paper is organized as follows. We present the existing problem and the proposed solution in Section 2. Section 3 provides measurements in real traffic conditions and methodology, followed by two different filtering approaches presented in Section 4 and Section 5. Results, analysis, and performance evaluation of the algorithm in terms of accuracy are presented in Section 6, followed by Section 7, concluding the paper.

## 2. Problem Formulation and Proposed Solution

The main objective of the C-ITS services is to enable new and enhanced automotive use cases for improving road safety, reliability, efficiency, and make them environmentally acceptable.

One of the most important use cases is considered driving inside of a tunnel environment. As explained above, by the time a vehicle enters a tunnel, the GNSS data are not available anymore, so the vehicle can not be localized. Since vehicle positioning is the central enabler for C-ITS services and applications, it is important to identify and develop new techniques to address this problem.

As an answer to this, we analyzed the potential use of communication between vehicles and the tunnel infrastructure concretely, we used our previous analysis on the V2I communication channel in the frequency band of the LTE-V radio interface [42] and designed an in-tunnel localization algorithm that makes use of the existing C-ITS infrastructure, at the same time it can be used by C-ITS for location estimation. Therefore, V2I RF signals are used for the location estimation of the vehicle.

C-ITS uses different technologies to enable communication between vehicles driving on the road (Vehicle-to-Vehicle, V2V), vehicles with traffic signals and roadside infrastructure (Vehicle-to-Infrastructure, V2I and I2V), as well as with other road users. This communication is used to share required awareness information related to each other’s position, dynamics, and attributes [43]. The exchanged cooperative awareness information is periodically transmitted as the Cooperative Awareness Message (CAM). According to the standard, the CAM message format contains a header and a body. The CAM includes information about the version, message identifier, generation time, ITS station ID and type (mobile, public authority, private, etc.), reference position information (latitude, longitude, elevation, and heading), and other accompanying CAM parameters, which can be optionally included [43].

In addition to this, C-ITS defines the Decentralized Environmental Notification Message (DENM) [44]. The periodic transmission of DENM messages over a specified area can be used to provide notification information about the relevant environment or traffic events, or road traffic conditions. This message, similar to CAM, contains a header and a body. The DENM collects information about the version, message identifier and generation time, the event type (e.g., vehicle breakdown, emergency situation, traffic jam), geographical position of the event, destination area indicating the geographical area where the DENM should be transmitted, transmission frequency of the DENM, etc. DENMs are disseminated to a longer distance than CAMs.

Currently, all related information to geographical vehicle positions and areas of interest are obtained using GNSS and IMU, which, as indicated above, are not available and accurate in tunnel environments. In this regard, we see the use of existing communication between V2I as a potential solution.

The construction, management, and processing of CAMs and DENMs are performed by the Cooperative Awareness basic service (CA basic service) and are easily extendable for the support of other types of C-ITS services and future C-ITS applications [43]. Therefore, we see these messages as potential candidates for the tunnel use case. In this work, we assume to use DENM to notify vehicles about the presence of a tunnel on the road and CAM to have communication between vehicles. Vehicles as the first C-ITS stations are referred to as On-board Units (OBU), and road infrastructure within the tunnel as the second C-ITS station is referred to as Road Side Unit (RSU). This existing communication is used to know the position of the vehicles, their behavior, and to monitor their speed.

### Vehicle Localization Algorithm Assumptions and Workflow

The main idea of the proposed localization algorithm is to combine the input parameters obtained using RF measurements and GNSS data to have the initial vehicle position in the area in front of the tunnel where the required GNSS signal strength is available. Then, when the vehicle continues towards the tunnel, the localization algorithm relies on RF measurements to continue providing position information in the areas where the GNSS signal is not available anymore.

When the vehicle receives the first DENM notification indicating the presence of a tunnel on the road, the vehicle system initiates the use of the localization algorithm, which is used in a tunnel scenario. The localization algorithm runs within the vehicle and is based on the following assumptions:

- The vehicle is moving in the defined lanes and has the OBU installed.
- When it enters the tunnel, there is no GNSS coverage, and the only communication technology in place is the technology provided by the RSU.
- The position of the RSU, its IP address, together with the ID, are continuously updated in the network. The network broadcasts this information to all vehicles driving in the tunnel’s surrounding area using DENM notifications, indicating the tunnel’s presence on the road.
- The received data by the application is authentic and includes security-related requirements.

The workflow of the steps that are considered for in-tunnel scenario location estimation is presented in Algorithm 1. As seen in Algorithm 1:

1. If the OBU system receives a DEMN notification pointing towards a tunnel environment, the OBU system initiates the tunnel localization application, which starts to collect and analyze the input data.
2. The tunnel localization application extracts the IP address, the ID, and the geo-location of the RSU placed within the tunnel in which it will have to hand over and continue V2I communication.
3. While the vehicle moves forward into the area covered by the network or RSU used in the tunnel environment, the vehicle requests to join the network covered by the RSU placed within the tunnel, which is assumed to cover the area at the entering zone of the tunnel.
4. The vehicle sends a CAM notification to share its ID and the initialization of the in-tunnel localization algorithm.
5. After successfully joining this network, the vehicle uses the localization application timer. For every timestamp defined on the application, it obtains the RF signals parameters and its current geo-location provided by GNSS.
6. Here the localization algorithm initiates the KF, explained in Section 4 and Section 5. The position obtained using the GNSS and RF propagation signals is used to know the initial position of the vehicle.
7. The current position of the vehicle is saved in the algorithm database.
8. In the next received frame from the RSU (depending on the input frequency), the vehicle receives a signal from which it extracts the RF channel information and measures the required RF parameters explained in Section 3. The RF parameters together with the initial position are used for position estimation, and this new position is saved again in the database.
9. This algorithm continues to estimate and update the position of the vehicle until the vehicle arrives at the end of the tunnel and moves into the area outside the tunnel.
10. As soon as the vehicle passes the indoor tunnel environment and can rely on the GNSS coverage for positioning, the status of the event changes, the localization algorithm stops the timer and estimations, as it can send a CAM message notifying the RSU that the vehicle passed the tunnel and the event is canceled.

**Algorithm 1:** Vehicle localization algorithm workflow used in tunnels environments.
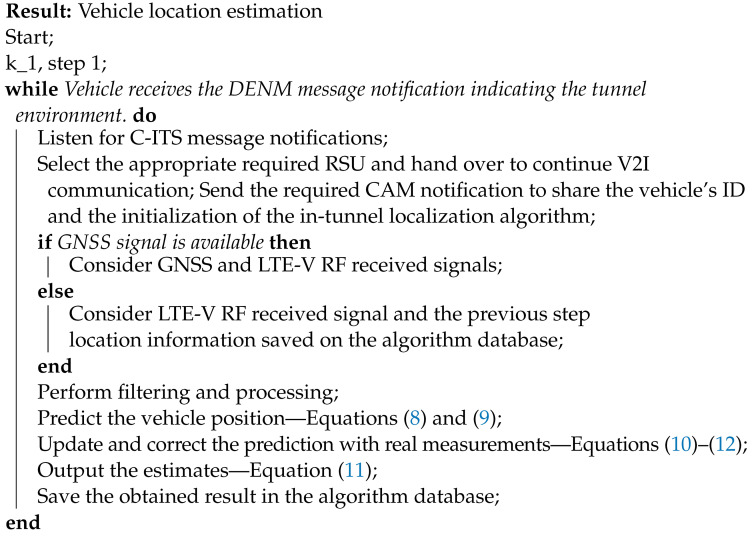


## 3. Measurements and Methodology

In this section, we present the channel measurements performed in a tunnel environment, followed by the analysis of the considered parameters for positioning using a vehicular communication system.

### 3.1. Measurement Setup and Scenario

**Figure 1:**
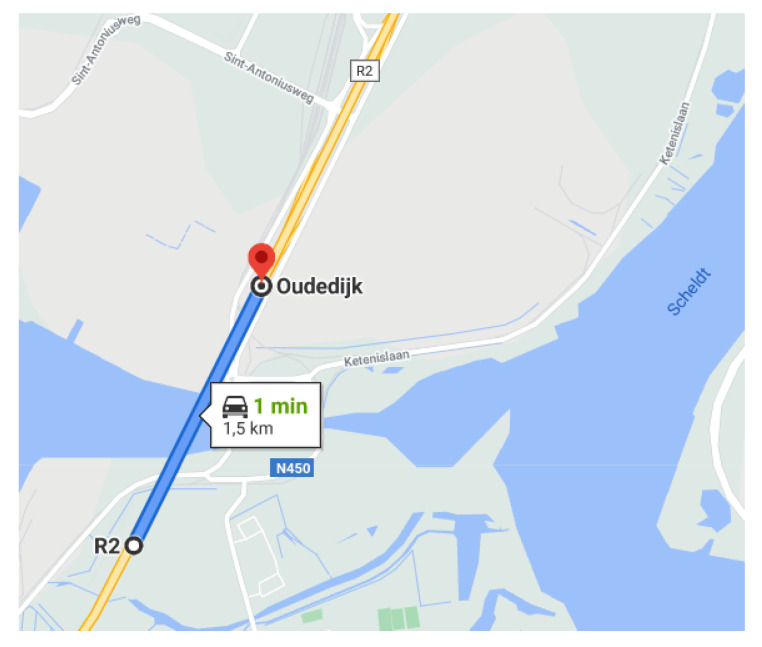
The Beveren tunnel in Antwerp, Belgium. The vehicle travels from point R2 with a speed of 90 km/h toward the end of the tunnel, marked with a teardrop-shaped location symbol. ©2021 Google.

As presented in our previous work [10], we performed channel measurements in the Beveren tunnel in Antwerp, Belgium. The measurement road segment starts at position R2 and continues until the end of the tunnel, marked with a teardrop-shaped location symbol. It covers the entire Beveren tunnel (see Figure 1). The tunnel has double-way roads with two rectangular tubes with a height of 5 m and a width of 10 m. Concrete blocks are placed on all sides of the tunnel together with lights and pipes.

The transmitter Tx considered as the C-ITS station was placed within the tunnel to ensure the V2I communication with the receiver Rx placed on the rooftop of a van carrying the Rx inside. The measurements were performed using the MIMOSA radio channel sounder [45], with an 80 MHz transmission bandwidth centered around a carrier frequency of 1.35 GHz. The carrier frequency lies conveniently within the operating band of the LTE-V standard [42] radio interface that supports V2I communications (named Uu-interface), which operates in the licensed 2 GHz band (880–2690 MHz) [10]. The channel sounder used for measurements provides samples of the continuous channel transfer function (CTF); that is, time-varying and frequency selective h(t;f). It identifies 819 frequency bins over B=80 MHz measured bandwidth for each snapshot, with a repetition time of ts=975.3 µs.

For this measurement campaign, a single wideband omnidirectional antenna was used at both Tx and Rx for the sounding signal, while we added 6 patch antennas at Rx just for synchronization and detection enhancement. The data from the Tx antenna was modulated onto the carrier using orthogonal frequency division multiplexing (OFDM). Table 1 summarizes the technical configuration of the MIMOSA channel sounder used for this measurement campaign [10].

As shown in our previous work and Figure 1, the vehicle speed through the tunnel was 90 km/h heading toward the Tx, then continuing on the second part of the tunnel, moving away from the transmitter. The total trip time was 54 s. The measurements were obtained using a snapshot repetition time of ts = 975.3 µs. We captured a number of samples in the frequency domain with a maximum Doppler shift of 1/2ts = 512 Hz and a minimum resolvable delay resolution of 12.5 ns. Following the approach presented in [46], we performed various runs, and the required parameters were stored every 0.8 m over the total considered segment.

### 3.2. Rf Propagation Analysis in Tunnels

Taking into consideration the fact that radio wave propagation in tunnel environments is different compared to outdoor and other indoor environments, tunnels are considered a complex environment for propagation analysis.

Tunnels behave as waveguides, which results in a longer communication range, but the observed fading is higher and non-stationary. There are no feasible analytical models to be used for electromagnetic wave propagation. Even in cases when the tunnel has very well-defined and simple geometries (rectangular or circular cross-section), there are no available solutions. Modal Theory and the Geometrical Optics Theory are the two most common approaches. In Modal Theory, tunnels are modeled as oversized imperfect waveguides with rectangular or circular geometry. The received field is the sum of the fields consisting of a fundamental mode and several higher-order modes [47,48]. However, considering the complexity and lack of flexibility of these approaches, they are not suitable for localization. Thus, we investigate the chances of using different RF propagation parameters to check if we can obtain acceptable localization results.

Therefore, we have used the information about varying statistical behavior for local regions defined by M=32 samples in time and N=819 samples in frequency and approximated as locally stationary. The approach is presented in the previous work [10]. As shown in [10], we constantly use the recorded Channel Transfer Function (CTF) to have the discrete CTF as $H\left[m;q\right]=H\left(t_{s}*m;f_{s}*q\right)$ and incorporate the concept of windowing on a defined frequency resolution $f_{s}=B/Q$, and time index $t_{s}$. The orthonormal 2-D tapering windows are computed from *K* and *L* orthogonal tapers in the time and frequency domains to have the discrete version of the local scattering function (LSF) multitaper-based estimator proposed in [49]. This process provides multiple independent spectral estimates from the measurements by estimating the spectrum of each individual taper. Averaging over timely tapered spectra gives the total estimated power spectrum, which is used to have the Doppler shift information.

#### 3.2.1. Doppler Shift

Doppler effect reflects the frequency shift of a radio wave due to the relative motion between the involved nodes (transmitter and receiver). This shift is directly proportional to the velocity and direction of the motion of the nodes with respect to the direction of arrival of the received wave [50,51]. If the node is moving in the direction of the transmitter node or away from it, the shift is positive or negative. The same approach is used to compute the user velocity by using the speed of satellites derived from the information contained in broadcast or precise ephemeris [52]. Moreover, radar and sonar technologies use the same signal characteristic for velocity estimation [53,54].

In our case, we use the Doppler shift caused by the downlink signal between the transmitter within the tunnel and receiver to estimate the speed of the receiver placed on the vehicle. As explained above, we use the LSF estimate per stationarity region to have the Doppler shift. Then, in order to decrease the ambiguity, noise, propagation losses, and other propagation effects, we use EKF and KF to improve the accuracy of the parameters.

#### 3.2.2. Time of Arrival, TOA

ToA obtained using RF signals is used to estimate the time interval needed for a signal to travel from one node to another [37]. The hardware in use, bandwidth, clocks synchronization, multipath conditions, and complexity of the surrounding radio propagation environment has a huge impact on the accuracy level, especially in harsh environments [55,56,57].

Vehicle position tracking using ToA of the LTE propagation signals has been analyzed in many studies [28,29,55,56,57,58]. Most of the studies use a number of LOS transmitter nodes and consider the difference between ToA measurements to perform the receiver node position estimation using multilateration or trilateration.

We use the CTF mentioned above to compute the corresponding discrete channel impulse response CIR. Then, ToA is obtained by considering the interval around the maximum of the CIR [28,59]. Since, in our case, we use a single transmitter or RSU, we apply the difference between successive ToA measurements, the difference between successive distances between Tx and Rx to have information about the traveling distance of the vehicle.

## 4. Extended Kalman Filter-Based In-Tunnel Tracking

The Doppler shift depends on the geometry between the transmitter and receiver, velocity vectors, and is exposed to noise and multipath interference. As a result, the representative equation that expresses the relation between the observed frequencies and velocities is highly nonlinear. To mitigate the effects of the channel and improve the positioning accuracy provided by the Doppler shift measurements, we use Extended Kalman Filter (EKF).

EKF is an extension of the KF to nonlinear filtering problems [60], while the KF is the closed-form solution to the Bayesian filtering equations for the filtering model, where the dynamic and measurement models are linear Gaussian [60]. Both filtering approaches are usually applied to model the systems that are characteristic of multiple inputs and output parameters, considering both stationary and non-stationary situations [61]. The filter consists of the initialization, prediction, and correction steps, for every input value, and it uses linear stochastic difference equations to estimate values of interest [62].

In our case study, the position and the velocity of the vehicle in Cartesian coordinates are represented by **p** = ${\left[x,y\right]}^{T}$ and **v** = ${\left[v_{x},v_{y}\right]}^{T}$, respectively. The vehicle drives towards the transmitter placed in **T** = ${\left[x_{t},y_{t}\right]}^{T}$, and maintains a distance known as ‖**p** − **T**‖. The state of the vehicle is given using position and velocity **s** = ${\left[p^{T},v^{T}\right]}^{T}$.

The system equation that describes the motion model of the vehicle in time is given in Equation (1). $s_{k}$ is a four-dimensional state variable **s** = ${\left[x,y,v_{x},v_{y}\right]}^{T}$, *F* is the state transition matrix (see Equation (2)), and $w_{k-1}$ is the process noise with a zero mean and *Q* covaraince (see Equation (3)). The spectral density of the process noise is *q* = 0.5.

(1) $$\begin{array}{c} s_{k}=Fs_{k-1}+w_{k-1} \end{array}.$$

(2) $$\begin{array}{c} F=\left[\begin{array}{cccc} 1 & 0 & dt & 0\\ 0 & 1 & 0 & dt\\ 0 & 0 & 1 & 0\\ 0 & 0 & 0 & 1 \end{array}\right] \end{array}.$$

(3) $$\begin{array}{c} Q=q\left[\begin{array}{cccc} dt^{3}/3 & 0 & dt^{2}/2 & 0\\ 0 & dt^{3}/3 & 0 & dt^{2}/2\\ dt^{2}/2 & 0 & dt & 0\\ 0 & dt^{2}/2 & 0 & dt \end{array}\right] \end{array}.$$

In order to control the behavior of the motion model, we use Doppler shift measurement data obtained on the current time step *k*. Here we add the measurement equation, which relates the Doppler shift measurement $z_{k}$ to the state $s_{k}$. $z_{k}$ is the measurement value corrupted by additive zero-mean noise $r_{k}$ with $R_{k}$ covariance caused by the environment (see Equation (4)).

(4) $$\begin{array}{c} z_{k}=h_{k}\left(s_{k}\right)+r_{k} \end{array}.$$

In the measurement model, $h_{k}\left(s_{k}\right)$ depends on the signal wavelength λ, the carrier frequency *f*, the speed of light within the tunnel medium, the vehicle velocity, and the position of the transmitter **T**[$x_{t},y_{t}$] (see Equation (5)).

(5) $$\begin{array}{c} h_{k}\left(s_{k}\right)=v_{k}\frac{f}{c}\frac{\left(s-T\right)}{\left\|s-T\right\|} \end{array}.$$

The measurement model is nonlinear; thus, we use EKF, which uses first-order linearization to turn a nonlinear problem into a linear one. According to [53], first-order linearization of the nonlinear Doppler function is assumed to be sufficient when it is possible to have some assumptions for the considered scenarios. In the tunnel scenario, this equation depends on the tunnel’s known physical limitations, the speed limit of the vehicles, an accurate initialization of variables, and covariance matrices information about the possible range of the required variables. Therefore, the measurement equation is linearized around the current target state $s_{k}$ using Taylor series expansion (see Equation (6)).

(6) $$\begin{array}{c} z_{k}=h_{k}\left(s_{k}\right)+r_{k}\approx h_{k}\left({\overset{ˇ}{s}}_{k}\right)+\frac{\partial h_{k}}{\partial s_{k}}\left(s_{k}-{\overset{ˇ}{s}}_{k}\right)+r_{k} \end{array}.$$

We neglect the second-order terms, so the standard KF equations can be applied for recursive state estimation. We use the Jacobian matrix $H_{k}$, which is the partial deviate of the first term in Equation (6), and it shows how fast each output of the function is changing along with each input value (see Equation (7)).

(7) $$\begin{array}{c} H_{k}=\frac{\partial h({\overset{ˇ}{s}}_{k})}{\partial s_{k}} \end{array}.$$

As stated above, the first step of the KF is the prediction phase, which predicts the current location of the vehicle based on the previous step and the process input. In our case, the initial value $s_{0}$, with a prior mean $m_{0}$, and covariance $P_{0}$, are provided by the GNSS. Then the prediction step predicts the current location and velocity ${\overset{ˇ}{s}}_{k}$ based on the previous step ${\hat{s}}_{k-1}$ and *F* (see Equation (8)). The predicted covariance matrix ${\overset{ˇ}{P}}_{k}$ depends on the former value of covariance ${\hat{P}}_{k-1}$, *F*, and $Q_{k-1}$ (see Equations (2) and (9)). In this work, the check symbol ($\overset{ˇ}{}$ ) above every parameter is used to indicate the prediction step results, while the hat symbol ( ^ ) shows the update/correction step results.

(8) $$\begin{array}{c} {\overset{ˇ}{s}}_{k}=F{\hat{s}}_{k-1} \end{array}.$$

(9) $$\begin{array}{c} {\overset{ˇ}{P}}_{k}=F{\hat{P}}_{k-1}F^{T}+Q_{k-1} \end{array}.$$

For the update step, we consider the measurements and follow Equations (10)–(12). The Kalman gain *K* expressed in Equation (10) determines to what extent the prediction ${\overset{ˇ}{s}}_{k}$ should be corrected at time step *k* to have the estimated value ${\hat{s}}_{k}$, Equation (11). This estimation/prediction error is the difference between the predicted value and the actual measurement. Depending on the value of covariance measurement noise $R_{k}$, this gain gives weight to the predicted or the measured value. A large value of $R_{k}$ results in a small *K*, which means that the predicted value does not reflect the measured one. On the contrary, if $R_{k}$ is small, it means that the measurements for the specific area are approximated with an insignificant error value. Matrix $H_{k}$ is expressed in Equation (7).

Note that $R_{k}$ is often assigned as a constant based on the measurements’ accuracy. However, in order to increase the accuracy, [63] suggests tuning the covariance by considering the difference between actual measurement and its estimated value using the information available at step *k*, Equations (13) and (14). While in [64], the use of the parameter *a* is introduced, which puts weights on previous estimates of *R*, and it causes less fluctuation of $R_{k}$.

We have considered constant and dynamic covariance values $R_{k}$ for the analyses performed in this work. In this work, the constant value of *R* is determined based on field measurements performed in the tunnel. In addition to this, we consider different values of *R* to analyze the impact the measurement variance has on the localization accuracy.

(10) $$\begin{array}{c} K_{k}={\overset{ˇ}{P}}_{k}H_{k}^{T}{\left(H_{k}{\overset{ˇ}{P}}_{k}H^{T}+R_{k}\right)}^{-1} \end{array}.$$

(11) $$\begin{array}{c} {\hat{s}}_{k}={\overset{ˇ}{s}}_{k}+K_{k}\left(y_{k}-h_{k}\left({\overset{ˇ}{s}}_{k}\right)\right) \end{array}.$$

(12) $$\begin{array}{c} {\hat{P}}_{k}=\left(I-K_{k}H_{k}\right){\overset{ˇ}{P}}_{k} \end{array}.$$

(13) $$\begin{array}{c} e_{k}=y_{k}-h_{k}\left({\hat{s}}_{k}\right) \end{array}.$$

(14) $$\begin{array}{c} R_{k}=E\left(e_{k}e_{k}^{T}\right)+H_{k}{\overset{ˇ}{P}}_{k}H_{k}^{T} \end{array}.$$

(15) $$\begin{array}{c} R_{k}=aR_{k-1}+\left(1-a\right)\left(e_{k}e_{k}^{T}+H_{k}{\overset{ˇ}{P}}_{k}H_{k}^{T}\right) \end{array}.$$

## 5. Kalman Filter-Based In-Tunnel Tracking

To further investigate the positioning accuracy in a tunnel environment, we examine a case in which we combined Doppler shift measurements and ToA measurements obtained from the same transmitter.

As shown above, in Section 3.2.2, ToA measurement or signal travel time indicates the distance between the Tx (or RSU) and Rx (or OBU). Thus, we perform the difference between two successive ToA measurements in a known and pre-defined time sample to obtain the movement information of the Rx.

The speed obtained from Doppler shift measurements and distance obtained from ToA measurements are estimated as two independent parameters and are used to have the movement and heading of the vehicle or Rx while it moves through the tunnel. In order to improve the algorithm performance and to limit the measurement ranges, we use Kalman Filter (KF) fed by ToA measurements and KF fed by Doppler shift measurements, respectively. The obtained results give us information on whether using a less complex method that uses KF gives us comparable or better results than the above-presented method, which uses EKF fed by Doppler shift measurements.

As in the previous case, for the motion model, we use the GNSS data to have the initial distance $d_{0}$ between Tx and Rx and the speed $v_{0}$ of the vehicle (Rx). This information is used to obtain the first error estimation and perform calibration when using travel time measurements. In addition to this, the shape of the tunnel, the in-tunnel waveguide signal propagation feature, and the defined angle range between Rx and Tx can be used to improve the accuracy.

(16) $$\begin{array}{c} x_{k}=x_{k-1}+\Delta r\sin\phi_{k-1}\\ y_{k}=y_{k-1}+\Delta r\cos\phi_{k-1} \end{array}.$$

In Equation (16), the position of the vehicle expressed using $x_{k}$ and $y_{k}$ depends on the previous position $x_{k-1}$ and $y_{k-1}$, the traveled distance or range Δr for the defined time interval *t*, and the vehicle dynamic heading $\phi_{k-1}$. The geometric range Δr is equal to the difference between two successive distances ($d_{k-1}$ and $d_{k}$) obtained from ToA measurements. The vehicle dynamic heading ϕ is approximately equal to its tangent to the trajectory curve when moving on the road [65]. Considering the fact that the dynamic heading of the vehicle cannot be changed abruptly as it moves on a road, since it has some limits, and adding here the fact that these heading limits become even more relevant within a tunnel environment, the heading of the vehicle can be determined using the historical trajectory curve. Therefore, in our case, we use the past estimated positions $x_{k-1}$ and $y_{k-1}$ of the vehicle to determine the upcoming heading for time or step *k*. The relationship between attitude and delta position and the followed approach is found in [65].

To have an accurate travel distance Δr that is the difference between two successive ToAs multiplied with the speed of light, we consider KF. In this case, the state vector ${\overset{ˇ}{s}}_{k}$ (17) is the distance between the transmitter and receiver. *F* is the state transition constant, which relates the present state ${\overset{ˇ}{s}}_{k}$ of the distance to its previous state ${\hat{s}}_{k-1}$. Since the distance parameter is a one-dimensional value, *F* is equal to 1.

(17) $$\begin{array}{c} {\overset{ˇ}{s}}_{k}=F{\hat{s}}_{k-1}\pm Bu_{k-1} \end{array}.$$

Parameter *B* in Equation (17) associates the control input parameter *u* to the distance value or state, thus *B* is the time interval considered for the next estimation, and *u* is the speed parameter. The sign in Equation (17) is “+” when the Rx is approaching the Tx so the Doppler shift is positive, and is “−” when the Doppler shift is negative. Further explanations about the inclusion of the Doppler shift in this model are given below.

In order to include the impact of noises and traffic changes, we consider the process noise covariance *Q* (*Q* = 0.5), thereby obtaining the covariance matrix ${\overset{ˇ}{P}}_{k}$, (see Equation (18)).

(18) $$\begin{array}{c} {\overset{ˇ}{P}}_{k}=F{\hat{P}}_{k-1}F^{T}+Q \end{array}.$$

ToA real data measurements $z_{k}$ are considered for the correction step, Equations (19)–(21). *H* is the transformation constant that relates the predicted state or distance ${\overset{ˇ}{s}}_{k}$ to measurements, and it is reciprocal of the speed of light H=(1/c). The obtained measurements include noise or uncertainty with a covariance value of *R*, which is determined based on the measurements performed in the tunnel. The Kalman gain *K*, expressed in Equation (19), has the same role as presented above and determines to what extent the predictions should be corrected in the time step *k*.

(19) $$\begin{array}{c} K_{k}=P_{k}H^{T}{\left(H{\overset{ˇ}{P}}_{k}H^{T}+R\right)}^{-1} \end{array}.$$

(20) $$\begin{array}{c} {\hat{s}}_{k}={\overset{ˇ}{s}}_{k}+K\left(z_{k}-H{\overset{ˇ}{s}}_{k}\right) \end{array}.$$

(21) $$\begin{array}{c} {\hat{P}}_{k}=\left(I-K_{k}H\right){\overset{ˇ}{P}}_{k} \end{array}.$$

The control input *u* in Equation (17) is the speed of the vehicle *v*, which, as previously mentioned, is estimated independently from the above ToA filtering process. For this parameter, we use KF fed by Doppler shift measurements. In this case, we ignore the nonlinearity considered in Section 4. To reduce redundancy in equations, we refer to the above general expressions of KF to explain the filtering and processing steps we followed for vehicle speed estimation.

We use the state vector ${\overset{ˇ}{s}}_{k}$ (17) to present the speed of the vehicle, while *F* is equal to 1 and relates the present state ${\overset{ˇ}{s}}_{k}$ of the speed to its previous state ${\hat{s}}_{k-1}$. Parameter *B* is equal to zero. Similarly, the impact of noises and traffic changes are presented using process noise covariance *Q* and covariance matrix ${\overset{ˇ}{P}}_{k}$ (see Equation (18)). We assume a constant *Q* (*Q* = 0.5) and initiate a $P_{0}$ value characteristic for the speed parameter.

We consider Doppler shift measurements for the correction step, Equations (19)–(21). *H* is the transformation constant (H=(f/c)), which relates the predicted state of the speed ${\overset{ˇ}{s}}_{k}$ with the measurements, while the covariance *R* is determined based on the Doppler shift measurements performed in the tunnel. The Kalman gain *K*, expressed in Equation (19), has the same role as presented above.

At the time $t_{0}$, the vehicle speed and distance are initiated using GNSS ($v_{0}$ and $d_{0}$). Then, for the next step, we use KF fed by Doppler shift measurements to estimate the speed parameter *v*. Further, this estimated speed value is included in the Equation (17) as the control input of the motion model to have the distance *d*, which is observed and filtered using KF fed by ToA measurements.

To compare the estimation accuracy of the speed values obtained using KF fed by Doppler shift measurements (presented in this section) and the speed values obtained using EKF fed by Doppler shift measurements (presented in Section 4), we investigate the third case when we use KF on ToA measurements for the heading estimation and EKF on Doppler shift measurements for speed estimation.

## 6. Results and Analysis

The main purpose of this work was to evaluate and analyze the performance of the in-tunnel vehicle localization algorithm in terms of the provided accuracy. We use the estimation error to obtain the required information about the accuracy. The estimation error is defined as the difference between the true trajectory followed by the vehicle and the resulting trajectory obtained using three different approaches. When the location estimate is performed using EKF fed by Doppler shift measurements (as described in Section 4), we consider this as the first approach and we referred to it as EKF DS. When using KF on individual Doppler shift and ToA measurements (as described in Section 5), we consider this as the second approach, and we refer to it as KF DS and ToA. The third approach is considered as the combination of the previous two, and we referred to it as EKF DS and KF ToA.

For the performance analysis, we examine the minimum, maximum, average, and standard deviation of the estimation error. Furthermore, we use the Root Mean Square Error (RMSE) to include information for the most significant estimation errors or higher deviations from the observed values. By considering these indicators, we have a complete picture of the estimation error distribution while the vehicle is driving inside the tunnel. In addition to this, we consider different values of measurement noise covariance to observe and monitor the algorithm dependency on the V2I channel measurements and the considered motion model.

**Figure 2:**
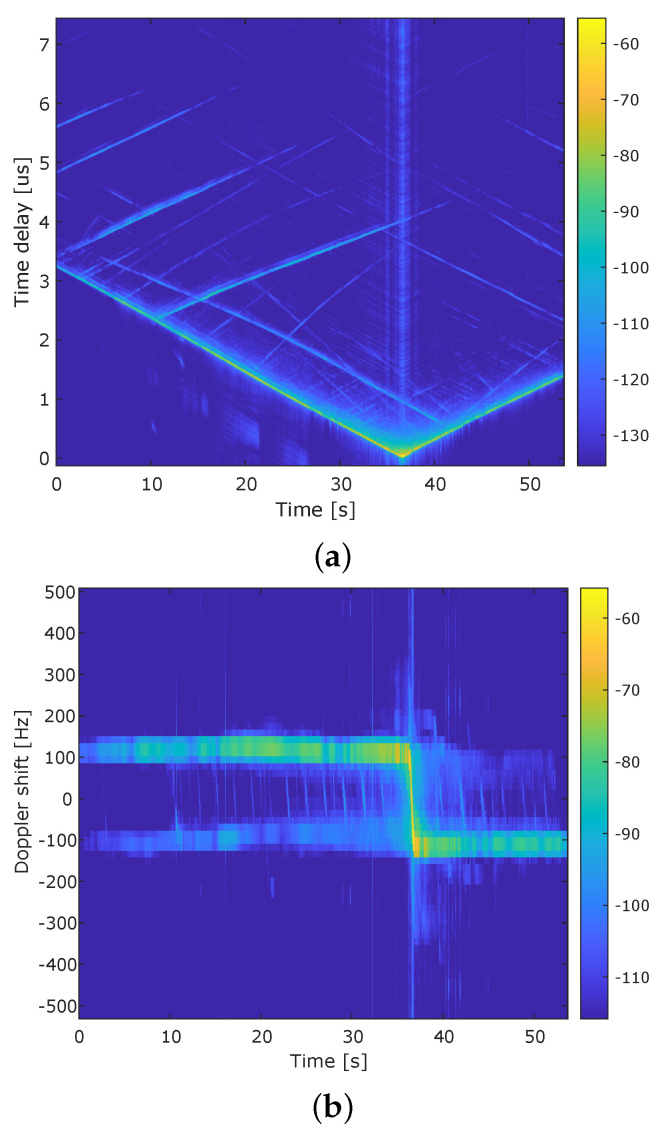
Time-varying (**a**) Power Delay Profile (PDP) and (**b**) Doppler Power Profile (DPP) for the scenario of crossing Tx at the known position with a constant speed of 90 km/h [10].

The V2I channel measurements performed in the Beveren tunnel in Antwerp are shown in Figure 2. Figure 2 shows the Power Delay Profile (PDP) and Doppler Power Profile (DPP). These results are obtained when integrating the LSF over the Doppler and delay domains [10]. As noticed in the figure, the delay decreases as Rx approaches Tx with a positive Doppler shift, then after crossing the Tx position, the delay starts increasing again, and the Doppler shift becomes negative. In both figures, several multipath components from fixed scatterers can be observed where the received power is higher (the part where Doppler shifts is between +/−100) and the components resulting from moving scatterers in the same movement direction have different Doppler shifts that are more consistent, depending on their relative speed and position [10].

The maximum measured Doppler shift is used to obtain the speed of the vehicle and then, as indicated above, to know the position of the vehicle. Moreover, ToA is used to know the distance between Tx and Rx and then use the difference between successive distances to know the road segment passed by the vehicle.

**Figure 3:**
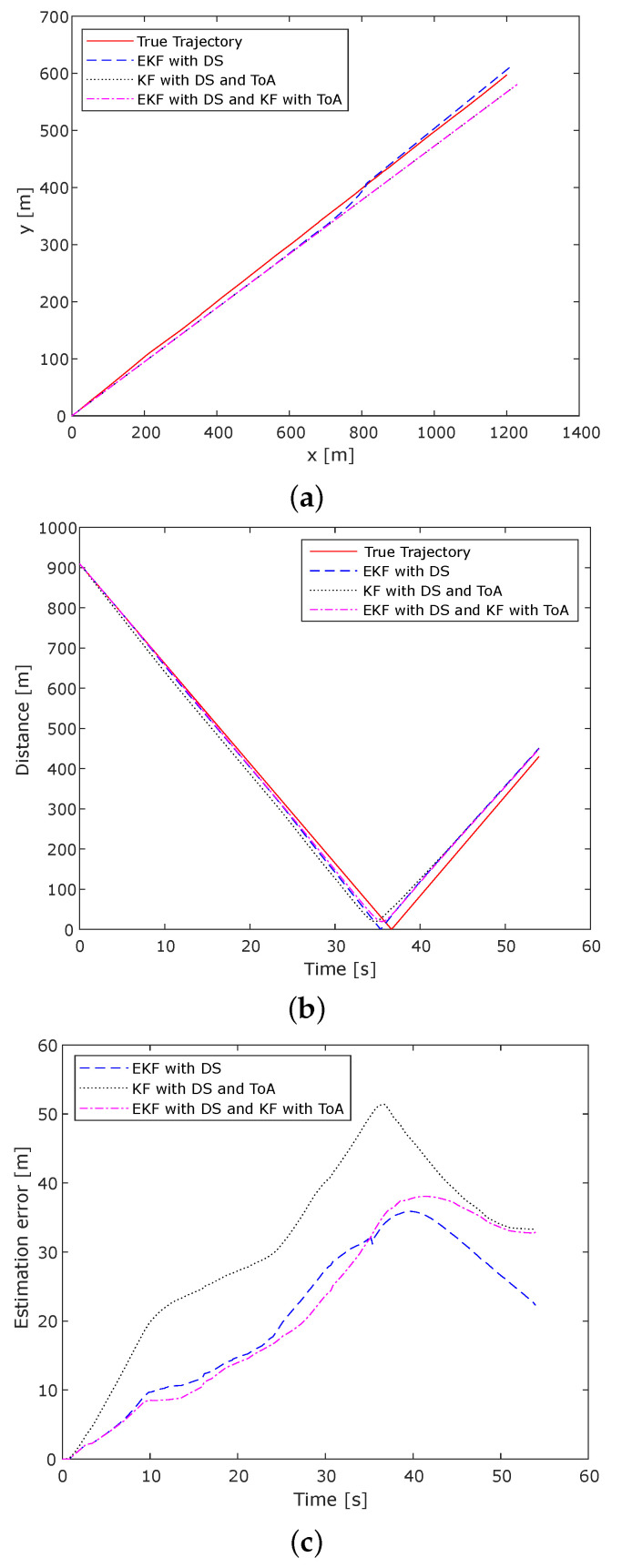
(**a**) True and estimated vehicle trajectory, (**b**) Distance between transmitter and receiver, and (**c**) Estimation error, when using Extended Kalman Filter on Doppler measurements (EKF DS), Kalman Filter on individual time and Doppler measurements (KF DS and ToA), and the combination of two previous approaches (EKF DS and KF ToA), while the vehicle is driving in the tunnel environment.

Figure 3 shows the difference between the estimated positions and the true positions of the vehicle while traveling in the tunnel environment. In Figure 3a the true trajectory results are compared with the ones obtained using EKF DS, KF DS and ToA, and the combination of EKF DS and KF ToA, on the xy-plane. These xy-plane values are a transform of the geodetic coordinates specified by latitudes, longitudes, and height to the local north, east, and down Cartesian coordinates specified by *x*North, *y*East, and *z*Down.

The calculated distance between Rx and Tx is shown in Figure 3b. The results in Figure 3b are related to Figure 2. While the vehicle approaches the transmitter near the second 37, the distance between Tx and Rx becomes shorter, and the number of scatters and noise is lower too, so we find higher accuracy for the speed and velocity estimation. In this part, the Doppler shift provides better results for the required parameters, similar to how the ToA estimation provides better accuracy. Therefore, the error tends to decrease (see Figure 3c); however, the cumulative error of the vehicle moving toward the Tx has an impact on the resulted estimation error in this part of the journey. When the vehicle Rx passes the Tx and continues its drive toward the exit of the tunnel, the distance between Rx and Tx increases, while the estimation error tends to decrease; however, the cumulative error on the continually existing Tx–Rx driving distance has its impact.

The estimation error is shown in Figure 3c. As we can see at the entrance of the tunnel, the vehicle position is known and is considered as the initial position, so the error is a minimum of 0 m for all three approaches used in the comparison study. Moving towards the tunnel, the error increases because of several multipath components from scatterers, NLOS conditions, traffic density, and accumulated error on existing Tx-Rx driving distance, reaching the maximum value of 35.88 m when EKF DS is used, 51.39 m when KF DS and ToA are used, and 38.06 m when EKF DS and KF ToA are used.

Comparing the results obtained when using EKF DS, KF DS, and ToA, and their combination as EKF DS and KF ToA shows that the EKF DS provides better accuracy. The EKF DS estimation errors values vary between 0 and 35.88 m. The mean value is 20.30 m and the standard deviation is 11.04 m. While for the case when KF is used on ToA and Doppler shift measurements (KF DS and ToA), the estimation error values vary between 0 (minimum value) and 51.39 m (maximum estimation error value). Its mean value is 30.46 m and the standard deviation is 13.27 m. In the third case (EKF DS and KF ToA), the error values vary between 0 and 38.06 m, while 21.04 m is the average value with a standard deviation of 12.66 m. Similar results were observed when comparing the RMSE for the three cases. When using EKF DS, the RMSE value is 23.11 m, while when using EKF DS and KF ToA the RMSE value is 24.55 m. The highest RMSE value is obtained when using KF DS and ToA, 33.22 m.

The results indicate that the EKF used on the measured Doppler shift within the tunnel can follow the movement of the vehicle better compared to KF when using Doppler shift and ToA individually. Numerical observations indicate that the estimation error obtained using EKF DS on average is 10 m lower than KF DS and ToA. The combination of EKF DS and KF ToA can improve the obtained accuracy by around 8 m on average. As expected, the positioning errors are higher if the positions are obtained using Doppler shift or ToA measurements without filtering methods. Thus, the obtained findings are not presented here. The identified problems are related to the RF multipath propagation, which is certainly a major source of positioning errors; the losses and NLOS conditions are caused by the surrounding environment.

On the proposed localization algorithm, multipath propagation and losses caused by the surrounding environment are modeled and considered on the location estimation. As indicated in Section 4 and Section 5, measurement trust-ability is defined using the covariance parameter *R*. Therefore, we have considered three different values of *R* useful for velocity estimation by using Doppler shift measurements. Moreover, we considered the dynamic *R*, as shown in Equation (15), to have the EKF on Doppler shift measurements.

**Figure 4:**
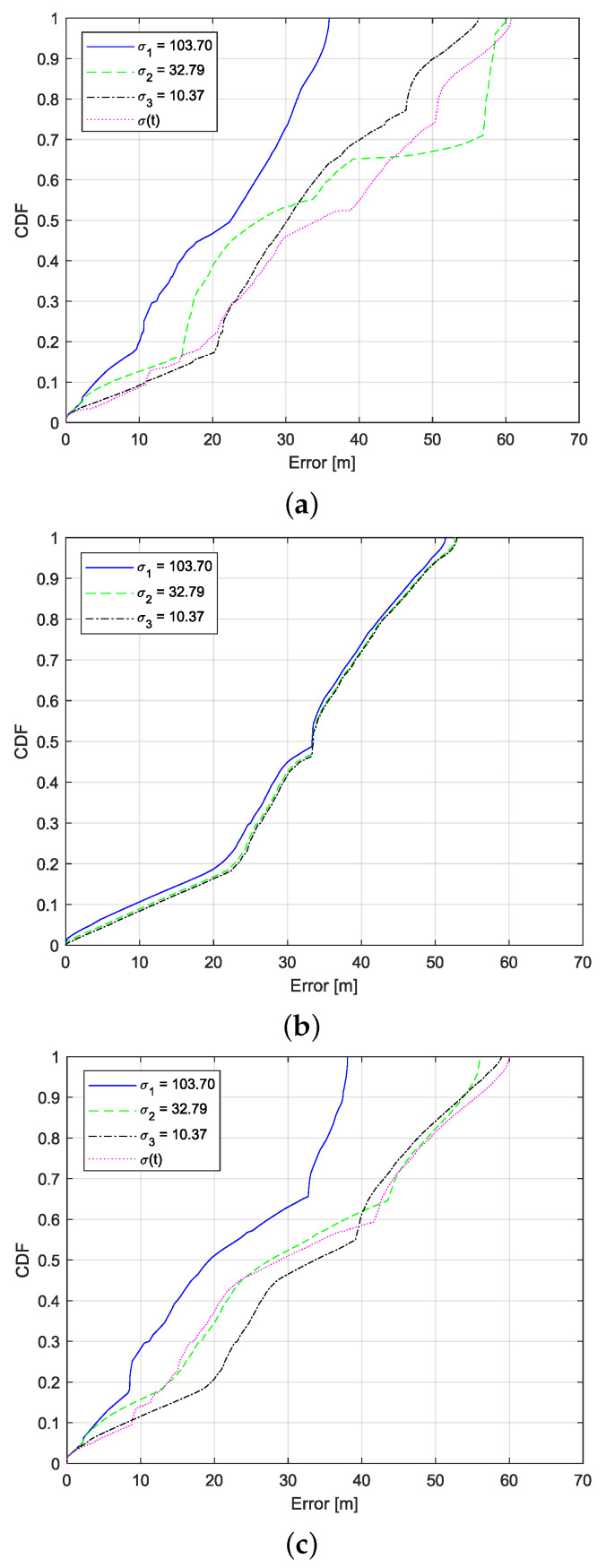
CDF of the location estimation errors for different filtering approaches (**a**) EKF DS, (**b**) KF DS and ToA, and (**c**) EKF DS and KF ToA, while using various standard deviation values $\sigma_{i}$.

Figure 4 shows the results of the Cumulative Distribution Function (CDF) of the estimation errors considering different noise covariance values expressed by standard deviation $\sigma_{i}$. The results imply that smaller noise covariance $R_{3}$ ($\sigma_{3}=10.37$ Hz) indicates a better channel environment, so the measurement error is lower, while the estimation error is higher since the motion model gives more trust to the measurement data. In such a situation, the value of the obtained error depends directly on the value of the measurement error. On the other hand, a higher value of *R* ($\sigma_{1}=103.70$ Hz), ($\sigma_{2}=32.79$ Hz) indicates that the measurements are distorted due to the multipath propagation effects. As a result, the algorithm using EKF and KF will trust the motion model more and the measurements less, so the estimation error is lower, see Figure 4 for all filtering approaches presented in this study.

When using EKF DS (see Figure 4a), 90% of the obtained errors are under 50.15 m when $\sigma_{3}$ is used, 58.19 m for $\sigma_{2}$, and 34.51 m for $\sigma_{1}$, respectively. When dynamic *R* is used, so the σ(t) depends on time, 90% of the estimation errors are under or equal to 55.98 m. In the second scenario, when using KF on DF and ToA, 90% of estimation errors are under 47.96 m for $\sigma_{3}$, 47.81 m when $\sigma_{2}$ is used, and 47.08 m when $\sigma_{1}$ is used (see Figure 4b). The same results were obtained when using EKF DS and KF on ToA. While increasing the $\sigma_{i}$ values, the estimation error decreases from 53.59 m for $\sigma_{3}$, to 53.75 m for $\sigma_{2}$, and 37.42 for $\sigma_{1}$. When using dynamic *R*, 90% of errors are under 55.53 m (see Figure 4c).

In terms of the achievable accuracy, our work differs from the previous studies in some key ways. Most of the studies [12,16,37] use a coverage range lower than 70 m, indicating the need for a higher number of transceivers depending on the technology and localization method, implying the need for LOS and presenting higher errors for respective distances between Tx and Rx. Therefore, we suggest combining the existing IMU vehicle system with C-ITS communication signals and improving in-tunnel environment RF propagation knowledge, so we can obtain the location estimation in tunnel environments where the GNSS signals are not reachable. The preliminary results indicate that the position estimation can be achieved without adding more hardware, which increases the C-ITS costs and causes the above-mentioned challenges.

The proposed algorithm solution can also be used for safety purposes since the C-ITS can monitor the behaviors of the vehicles in a tunnel environment as a complementary solution to the cameras or as the primary solution if cameras are not found. In addition to this, this solution can be used to indicate and monitor the vehicle’s speed when entering and driving through the tunnel and check if the drivers respect the required limits. This solution can reduce the radar application costs and solve the problem of not having radars everywhere inside tunnels and on highways. As a result, using the existing V2I communication infrastructure would reduce the cost and the need for different devices and technologies. This would have an impact on the efficiency and environment too.

## 7. Conclusions

In this work, we proposed a novel positioning algorithm based on Doppler shift and Time of Arrival measurements obtained using the existing V2I communication signals in tunnel environments, where GNSS signals are not available. The main objective is to contribute to the enhancement of vehicle localization solutions as location information is the central enabler for C-ITS services and applications.

The proposed positioning method employs the combination of the initial GNSS position and velocity of the vehicle driving into the tunnel and then uses the Doppler shift and Time of Arrival measurements to track the vehicle inside the tunnel. To improve the positioning accuracy, EKF and KF are introduced to mitigate the RF effects of path losses, channel noise, ambiguity, NLOS impacts, and multipath interference.

The results indicate that the EKF used on the measured Doppler shift (EKF DS) within the tunnel can follow the movement of the vehicle better compared to KF fed by Doppler shift and Time of Arrival measurements individually (KF DF and ToA). EKF DS provides an average estimation error 10 m lower than KF DS and ToA. Their combination, EKF DS and KF ToA, can improve the obtained accuracy results by around 8 m on average. As expected, the results are even worse if the position is obtained using Doppler shift or Time of Arrival measurements without filtering. The results show that an accurate value of measurement covariance can significantly improve the positioning accuracy since it provides a real picture of performed measurements as compared to a default covariance value.

In addition, the knowledge about RF propagation caused by the in-tunnel surrounding environment, the tunnel structure and dimensions, and traffic conditions on a timely/daily interval, and other known limits related to traffic, can further enhance the positioning accuracy.

The position and speed information provided by the proposed solution can monitor the vehicle behavior within the tunnel. Unlike existing positioning methods, the proposed one does not add more components and costs to the existing C-ITS infrastructure. As a result, the solution can further improve the safety, efficiency, and environmental requirements in traffic and transportation.

Future work will include angle of arrival analysis as a way to improve the current results. Furthermore, modeling in-tunnel propagation losses and fading considering NLOS identification can further improve the obtained results regarding speed, time, and position.

## Figures and Tables

**Table 1:** MIMOSA Channel Sounder Configuration.

Parameter	Setting
Bandwidth	80 MHz
Center frequency	1.35 GHz
Tx and Rx polarization	V (omni), V/H (patch)
Tx and Rx gain	2 dBi (omni), 7.4 dBi (patch)
Tx and Rx HPBW (patch)	$120^{\circ }$
OFDM symbol duration	81.92 µs
Minimum snapshot acquisition time	327.68 µs
Total recording time per trip	54 s
